# Endovascular Localization of Aortic Injury in a Porcine Model

**DOI:** 10.1109/OJEMB.2025.3556987

**Published:** 2025-04-02

**Authors:** Saaid H. Arshad, Ryan L. Touzjian, Matthew C. Jones, Brian A. Telfer, Jason M. Rall, Theodore G. Hart, Marlin W. Causey

**Affiliations:** Lincoln LaboratoryMassachusetts Institute of Technology 2167 Lexington MA 02421 USA; U.S. Air Force 59th Medical Wing Lackland TX 78236 USA

**Keywords:** Endovascular, hemorrhage, prehospital, prolonged field care, localization

## Abstract

*Goal*: Non-compressible torso hemorrhage represents a category of lethal injuries in both civilian and military traumatically injured populations that with proper intervention, training, or technological advancements are survivable. Endovascular localization of active bleeding in the pre-hospital setting can allow faster, less invasive, and more accurate applications of life-saving interventions. In this paper, we report initial in vivo and in silico experimental results to test the feasibility of endovascular localization of hemorrhage. *Methods:* Endovascular pressure waveforms were acquired on five pigs with an induced aortic injury via a custom intra-aortic catheter instrumented with four pressure sensors. Pressure and velocity data were then simulated on an in silico human aortic model with the same kind of injury. *Results:* A decrease in pulse pressure across the injury (proximal to distal) reliably indicated the injury location to within a few centimeters. The simulated model showed a similar decrease in pulse pressure as well as an increase in velocity*. Conclusions:* With additional refinement, localization accuracy may be sufficient for application of a modern covered stent to stop bleeding. The simulated model results indicate relevance for humans and provide guidance for future experiments.

## I. Introduction

Non-compressible torso hemorrhage is at the forefront of traumatic injuries because this injury pattern can be lethal without intervention, but survivable with proper training, technology and intervention in combat and civilian settings [1], [2], [3], [4], [5]. Early, pre-hospital identification and localization of active hemorrhage is critical to saving lives. The FAST exam (Focused Abdominal Sonography for Trauma) helps identify the presence of hemorrhage [6], [7], [8], [9]. However, the FAST exam does not identify active hemorrhage or localize the bleeding source [6], [7], [8], [9]. Localization methods that are used in the hospital, namely computed tomography (CT) and invasive open surgery [10], [11], are not options in the pre-hospital setting. Currently, approaches to pre-hospital interventions do not leverage or provide localization, namely endovascular aortic occlusion (REBOA) [12] or intracavity foam [13] or other intracavity agents [14]. However, if hemorrhage can be localized more accurately in the pre-hospital, then more localized interventions can be applied. Contrast-enhanced ultrasound [15] and endovascular sensing are two options for localizing hemorrhage. This is important because, if hemorrhage can be localized, then a number of standard endovascular interventions can be performed thereby minimizing invasiveness, morbidity, and likely mortality.

In this paper, we report on initial in vivo and in silico experiments to determine if torso hemorrhage can be localized via sensing from an intra-aortic catheter, inserted through the femoral artery as is done for REBOA. We measured endovascular pressure waveforms at 42 time windows of approximately 30s duration each, before and after creating a thoracic aorta hemorrhage in five pigs. We analyzed pressure-based features and developed an algorithm to locate the hemorrhage. We then modeled pressure and flow waveforms in a simulated human subject based on the measured physiology, in particular a vasospasm. We determined that the hemorrhage can be located to within a few centimeters along the aorta based on changes to pulse pressure.

Locating a hemorrhage in the femoral artery has been reported based on changes in the resistive index [16], [17] measured by noninvasive ultrasound, but the work reported herein is the first, to the authors’ knowledge, to evaluate endovascular sensing and localization of an aortic hemorrhage. We expect that these initial promising results for an aortic hemorrhage will point toward more generalized methods to localize torso hemorrhage to primary branch arteries and associated organs.

## II. Materials and Methods

The in vivo study is described in Section II-A, followed by signal processing of the in vivo data (Section II-B) and computational modeling (Section II-C). The supplementary material provides more details.

### A. In Vivo Porcine Hemorrhage Model

#### 1) Pressure Waveform Measurements

The experimental protocol and porcine hemorrhage model were developed by the United States Air Force 59th Medical Wing and approved by the local Institutional Animal Care and Use Committee. The study was conducted in accordance with the regulations and guidelines of the Animal Welfare Act and by following the National Research Council's Guide for the Care and Use of Laboratory Animals. Five female anesthetized Yorkshire swine (Sus scrofa, 82.6 ± 5.9 kg) were utilized (see Supplementary Section I-A). A laparotomy was performed and the abdominal and distal thoracic aorta was exposed, circumferentially dissected, and controlled to allow creation of a 3.6 mm injury, standardized through aortic punch biopsy device.

**Fig. 1.**
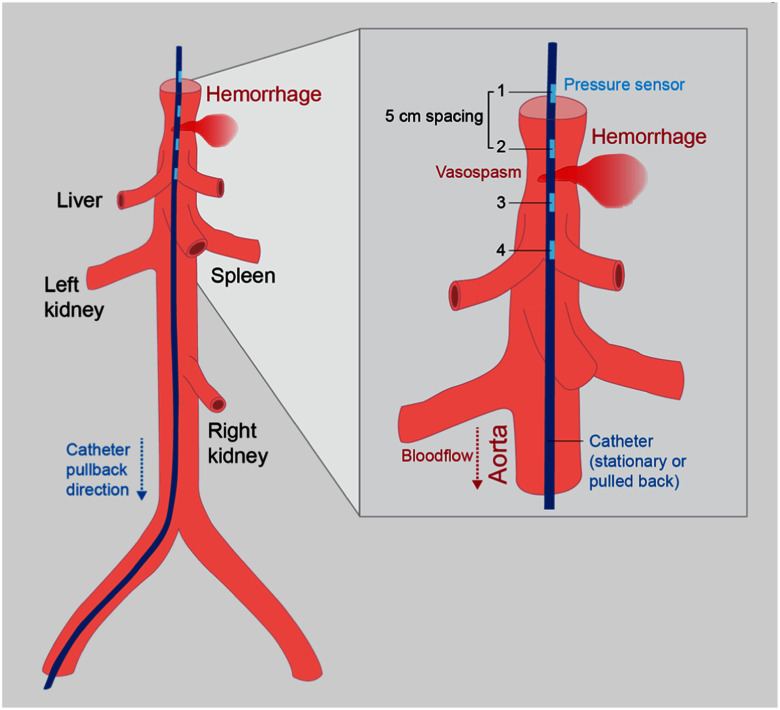
Illustration of the MSPC with 4 pressure sensors deployed in the aorta.

Endovascular pressure was measured with a custom multi-sensor pressure catheter (MSPC, Millar, Houston TX USA) with four sensors spaced 5 cm apart to allow simultaneous measurement at separate locations, as illustrated in Fig. 1. Before inducing the injury, the left femoral sheath was upsized and used to advance the MSPC so that sensors 1 and 2 were proximal and 3 and 4 were distal to the location of the planned injury. Pre-injury baseline pressure waveforms were measured. Pullback measurements were also recorded at baseline, with the catheter tip initially positioned near the subclavian artery and pulled distally at an estimated rate of 1 cm/s. This set of static and pullback measurements was then repeated after the injury multiple times to compare pressure waveforms and derived features. For the pullback measurements, the time was recorded (to nearest second) when the midpoint of sensors 2 and 3 passed the injury. Pressure data was sampled at 1 kHz. The pressure sensors were recalibrated before inserting the catheter for each pig. Baseline and post-injury data were then collected in a single continuous recording for each pig. While hemorrhage was being induced, the catheter was positioned within the external iliac artery sheath, protected from hemodynamic changes/effects.

#### 2) Aortic Diameter Measurements

An aortic vasospasm was observed at the injury location. The resulting reduction in aortic diameter and cross-sectional area was measured by intravascular ultrasound (IVUS, IntraSight Mobile, Philips, Cambridge, MA, USA). These measurements were performed on 15 separate swine of the same type and following the same protocol described in section A.1.

### B. Pressure Waveform Signal Processing and Analysis

The pressure waveforms were preprocessed and features extracted as described in previous work [18], [19], [20]. Key features extracted were peak-to-peak interval (PPI), systolic blood pressure (SBP), and pulse pressure (PP). Further details are in Supplementary Section I-B.

The effectiveness of each of these single features to differentiate between ABP waveforms proximal and distal to the injury was assessed with receiver operating characteristics (ROCs). For each feature, an ROC was generated across all subjects and a single decision threshold was determined. The analysis was repeated for each feature after normalizing by the maximum feature value observed during each individual pullback recording.

The pullback data represent the intended application of this work to localize hemorrhage. As the MSPC is pulled back through the aorta, a change in a feature value is hypothesized to indicate the location of the injury. The maximum point of negative change was found from the first derivative of the filtered feature vector. The point of injury was indicated as the time when the center point between sensors 2 and 3 passed the injury location. The timestamps indicated by the maximum feature change of each sensor were averaged to estimate the time at which the center point passed the injury.

### C. Computational Aorta Model

**Fig. 2.**
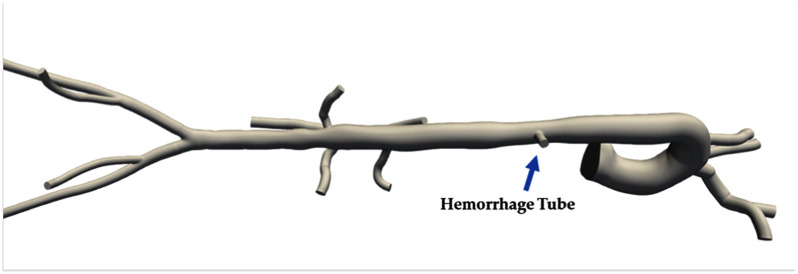
A visualization of the SimVascular aorta model with a vasospasm and tube added in the descending aorta to model an injury similar to those induced in the in vivo porcine experiments.

In addition to the porcine experiments, a computational human aorta model (Fig. 2) was developed to determine if the localization algorithm generalizes to a broader range of hemorrhage conditions and to humans. The computational model also provides flow waveforms, in addition to pressure, and so can be used to inform future in vivo experiments with additional sensors.

The computational model uses the SimVascular software package [22], with the aorta model depicted in Fig. 2. An uninjured baseline result was computed, as well as hemorrhage rates of 50, 100, 200, and 800 ml/min. Pressure and velocity measurements were sampled along the center-line of the aorta starting from the inlet into the ascending aorta. PP and end-systolic velocity (ESV) were extracted as features from the pressure and velocity waveforms, respectively. Pressure and velocity waveforms and derived features were computed at approximately 0.5 cm spacing in the aorta, generating a simulated pullback result to compare with the in vivo pullback results. Further details on the parameters used for the simulation can be found in supplementary Section I-C.

## III. Results

Porcine results are reported in Section III-A and III-B. Computational modeling results are reported in Section III-C.

### A. Pressure Change Analysis

The porcine characteristics are summarized in supplementary Tables III and IV, including time from injury to end of surgery and blood loss. All subjects had pre-injury baseline and post-injury pullback data for comparison. Subjects 1625 and 1626 did not have pre-injury baseline static measurements due to experimental error. A total of 42 measurements (time windows) were collected for the five pigs, with each measurement approximately 30s in duration.

**Fig. 3.**
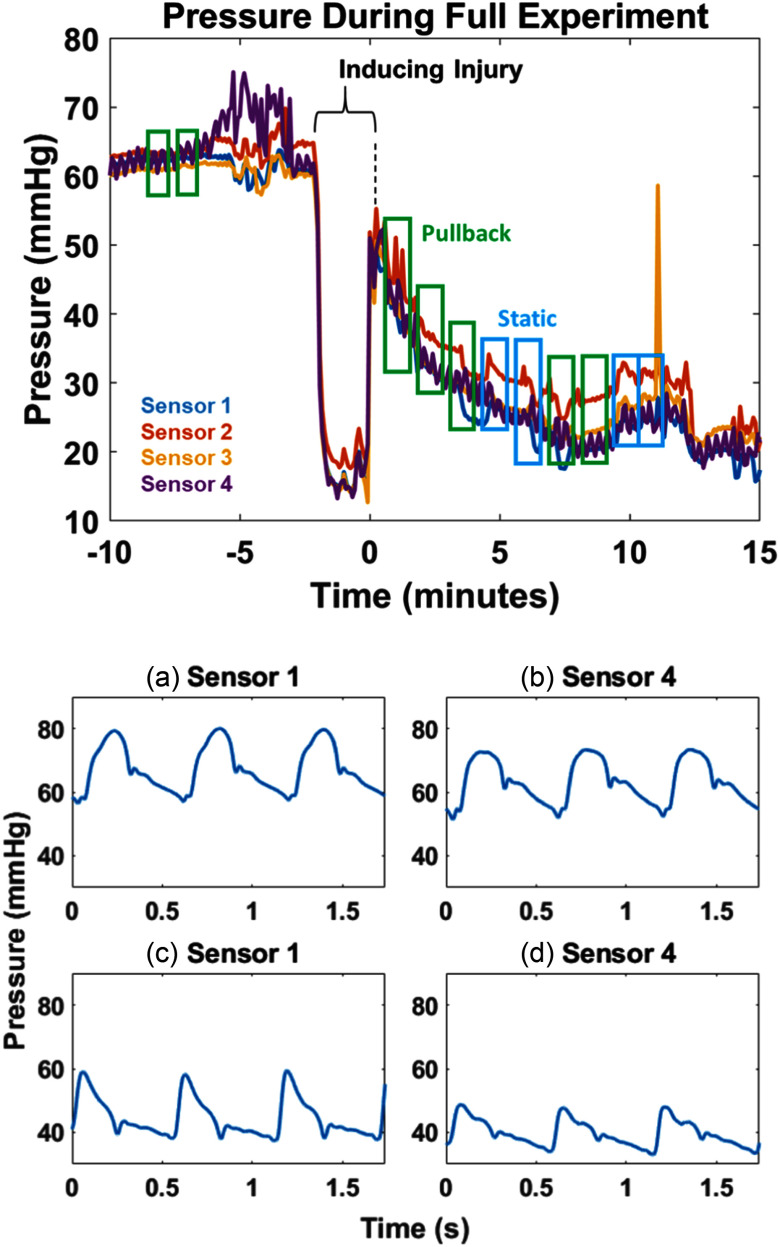
(above) Pressure traces from subject 1626 for the four MSPC sensors across the experiment, showing baseline measurements (-10 to ∼-4 minutes), injury being induced (∼4 to 0 minutes), and injury measurements after 0 minutes. (below) Pressure traces of three pulses from Subject 1654 for (a) pre-injury at sensor 1, (b) pre-injury at sensor 4, (c) post-injury at sensor 1, positioned proximal of the injury, and (d) post-injury at sensor 4, positioned distal of the injury.

Fig. 3 provides an overview of the experimental timeline with the four pressure sensors for an example subject. The injury phase starts at zero seconds. Four static experiments and seven pullback experiments were conducted on this subject. Fig. 3 also shows example pressure traces at sensors 1 and 4 taken before and after the injury during a static measurement. They illustrate the changes in waveform morphology and amplitude caused by the injury.

The ROC analysis determined that the pressure-based features, namely PP and SBP, had the highest areas-under-the-curve (AUCs) before and after normalization (Supplementary Section Table V). Since PP is the best-performing single feature with an AUC of 0.93 (0.95 after normalization), the following analysis focuses on PP.

**Fig. 4.**
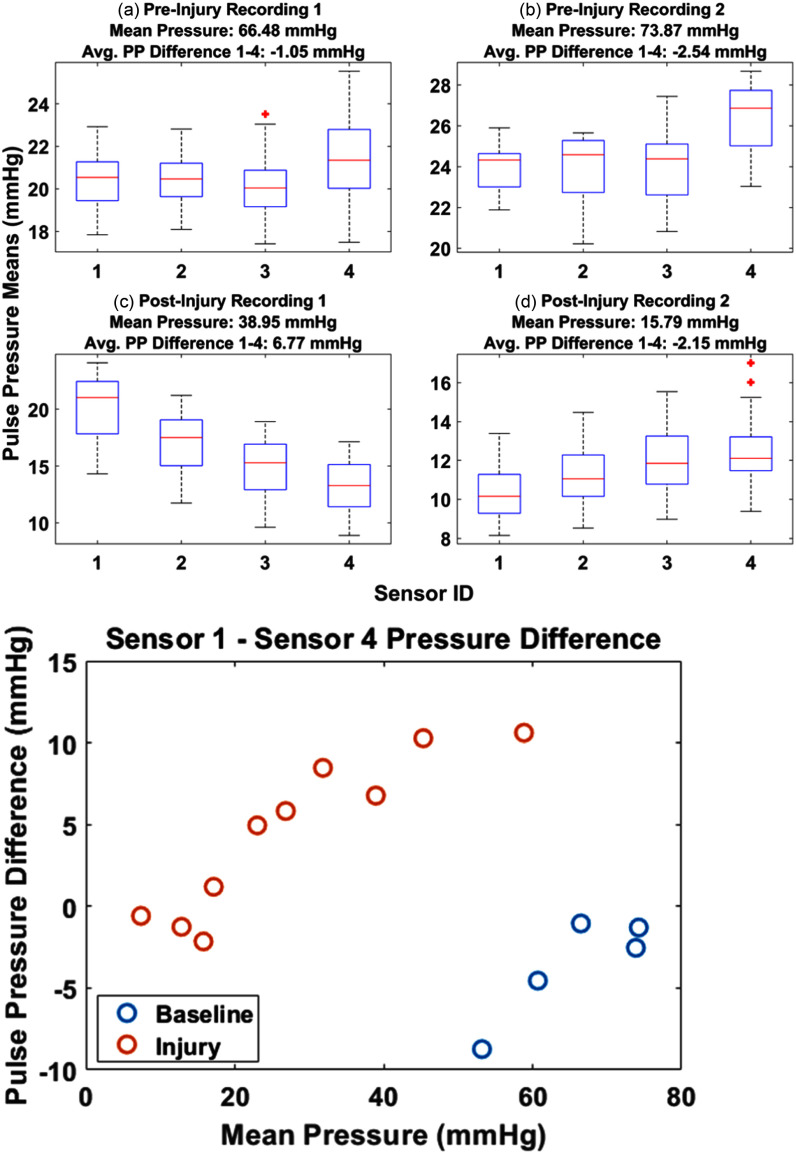
(above) Static pressure measurements at the injury location in subject 1654 before and after the injury is induced. The distal sensors (3 and 4) show pulse pressures greater than or equal to the proximal sensors (1 and 2) in the baseline cases (a) and (b). The higher mean ABP (i.e., more blood volume), the more pronounced the PP difference between sensor 1 and 4. When the mean ABP pressure is less than 20 mmHg the pulse pressure difference becomes negligible. (below) The static baseline (Fig. 3(c)) average pulse pressure differences between sensor 1 and 4 are plotted relative to mean ABP.

As shown in Fig. 4, the static pre-injury measurements for proximal PPs (sensors 1 and 2) are similar to or less than the distal PPs (sensors 3 and 4). This contrasts with the static post-injury measurements, for which the proximal PPs tend to be larger than the distal PPs. The proximal-to-distal PP difference becomes small in Fig. 4(d) because the mean ABP is low, less than 20 mmHg. The correlation in PP difference with mean ABP is apparent in the Fig. 4 scatterplot. The Pearson correlation is 0.91 (p < 0.05), for the injury data.

**Fig. 5.**
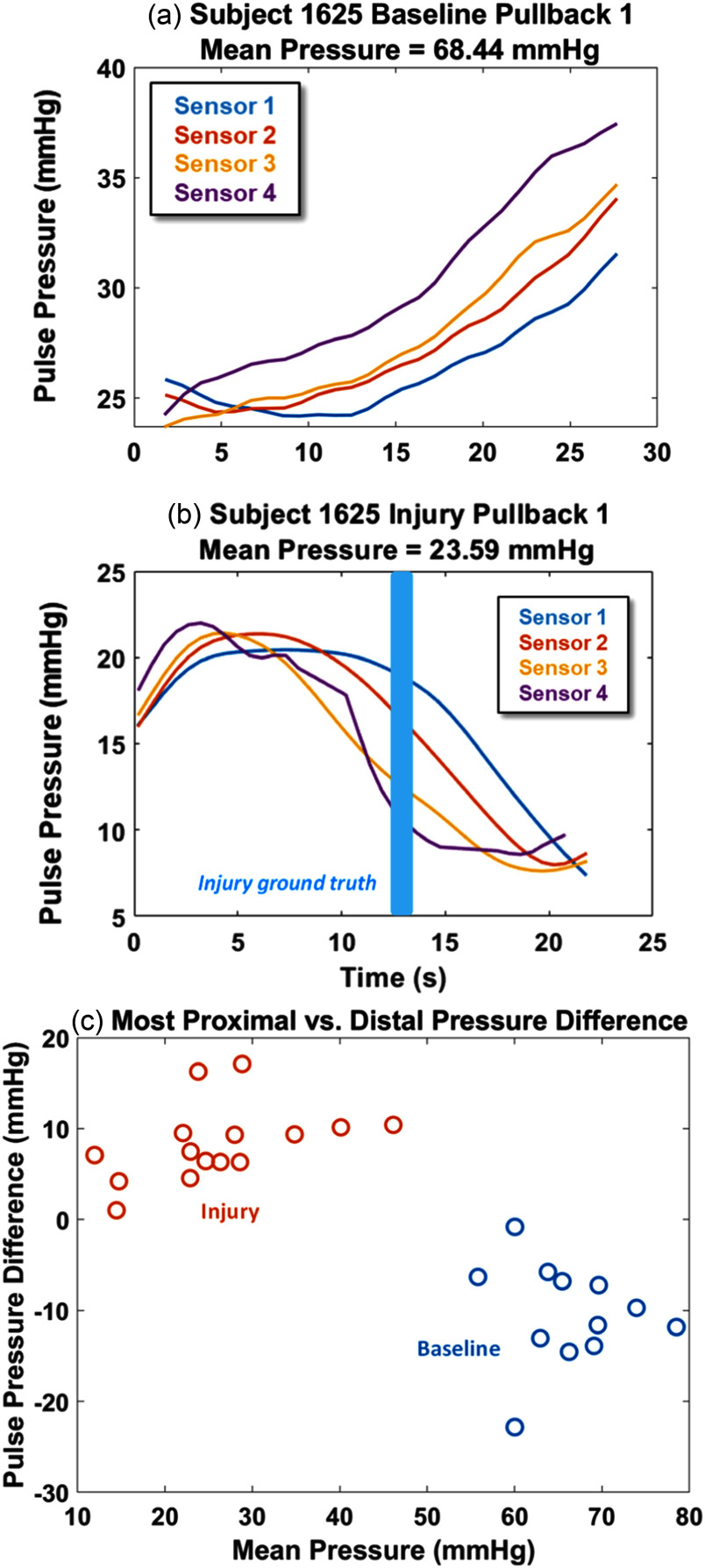
The (a) baseline pullback and (b) injury pullback data is shown for all sensors for subject 1625. The baseline pullbacks show an average increase of PP as the catheter is moved distally whereas the injury pullbacks show a significant decrease, with the highest point of change occurring near the point of injury, indicated by light blue rectangle. (c) Mean ABP and PP changes from injury and baseline pullback experiments. The PP difference reported is the difference between the most distal point recorded by sensor 4 and the most proximal point recorded by sensor 1.

Consistent with the static measurements, the baseline pullback measurements show an average increase in the PP as the catheter is moved distally, whereas the injury pull-back measurements show an average decrease. Fig. 5 shows an example of a baseline and injury pullback, with a high-magnitude negative slope occurring as the center of the catheter passes the point of injury. Fig. 5(c) shows the magnitude of the maximum PP decrease, calculated as the PP difference between the most distal point recorded by sensor 4 and the most proximal point recorded by sensor 1, plotted against the mean pressure over the respective recording. Pearson correlation coefficient of mean ABP and PP change for the injury data (red circles) is 0.76 (p < 0.05).

### B. Localization Algorithm

**Fig. 6.**
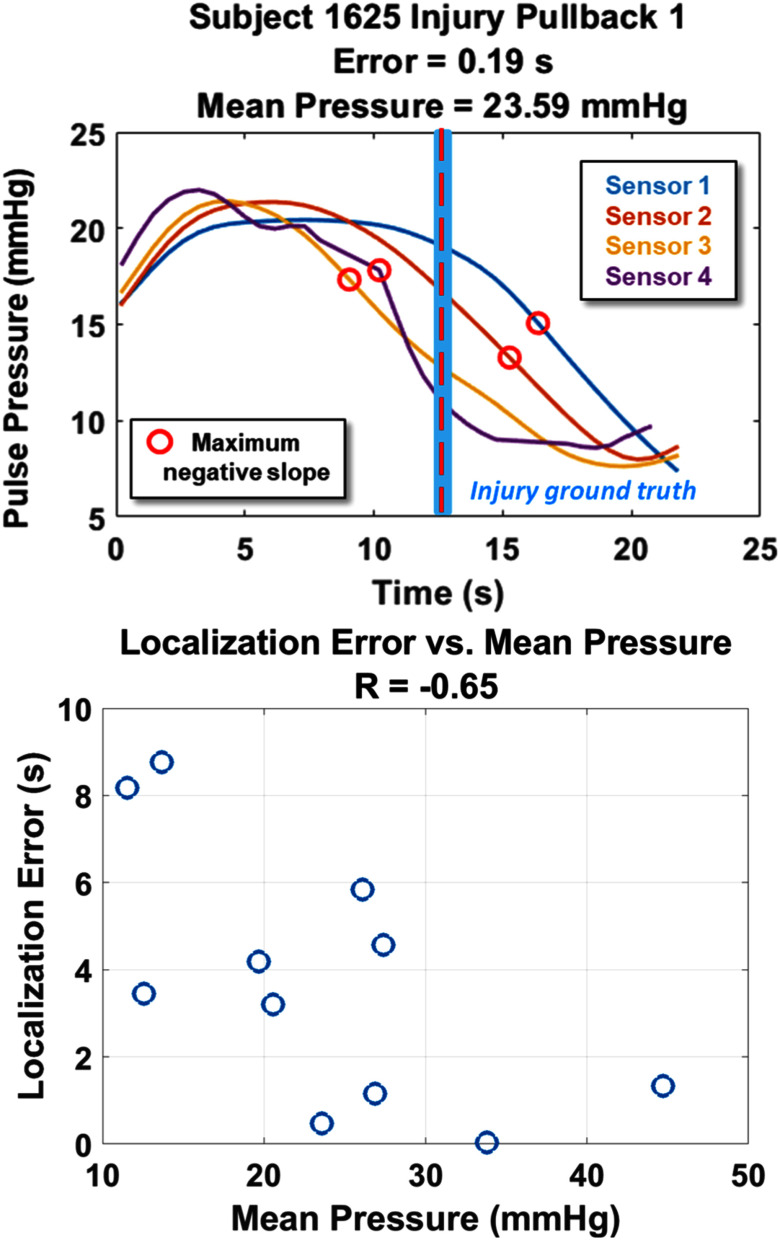
(a) Localization algorithm example. The light blue rectangle indicates the time stamp at which the catheter passed the point of injury. The red circles are the maximum negative derivatives on the filtered PP curves, and the dashed red line is the averaged final estimate of the injury location based on the estimates from each sensor. (b) The absolute value of the localization error is plotted relative to mean pressure. The accuracy of the localization is dependent on the mean pressure.

All baseline and injury pullback data were analyzed. All baseline data were rejected as having no hemorrhage because of increases (rather than decreases) in pulse pressure. Fig. 6 shows the results of the localization using the maximum negative derivative on the filtered PP curve. The average signed error (i.e., bias) excluding recordings with MAP less than 20 mmHg was 0.05 ± 3.5 seconds. Fig. 6(b) shows the relationship of the localization error with the mean pressure.

### C. Computational Model Results

**Fig. 7.**
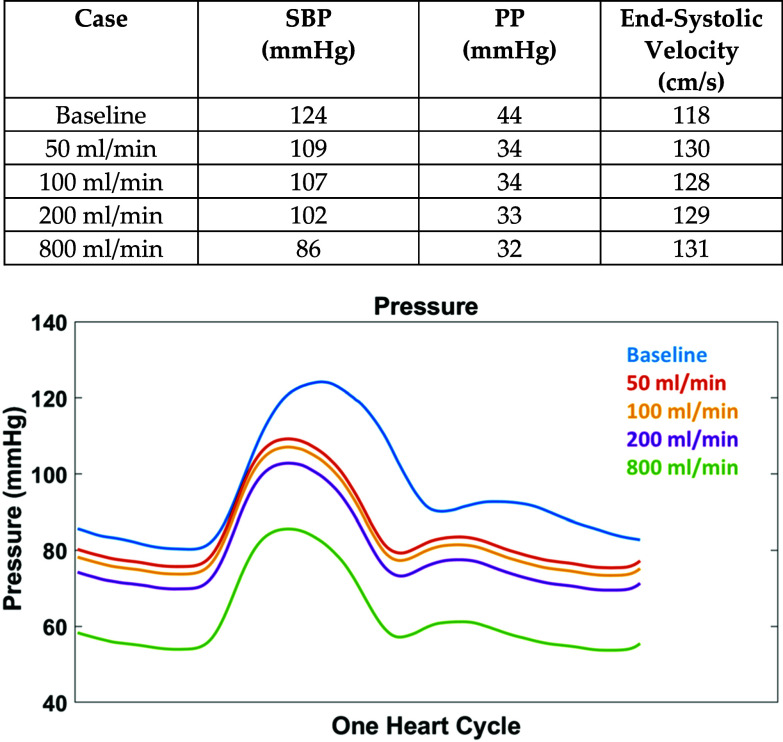
(table) Maximum pressure and velocity values shown for the waveforms below. (plot) A comparison of pressure at baseline and the four simulated bleeding rates at a single point in the aorta near the injury location from a single heartbeat.

The in vivo porcine results show that the signal of interest for localization is a PP decrease when moving distally across the injury site. A combination of the pressure changes due to blood loss, coupled with the body's vasospasm response, contribute to this signal. These phenomena were modeled in silico for a human aorta to assess relevance for humans. The pressure waveforms at a point in the aorta at the centerline are shown in Fig. 7. The simulated pressure waveform falls in the expected range of values for baseline and injury but lacks the complexity of the in vivo waveforms. Fig. 7 tabulates the pressure and velocity values. SBP decreases with larger bleeding rates while ESV is less impacted.

**Fig. 8.**
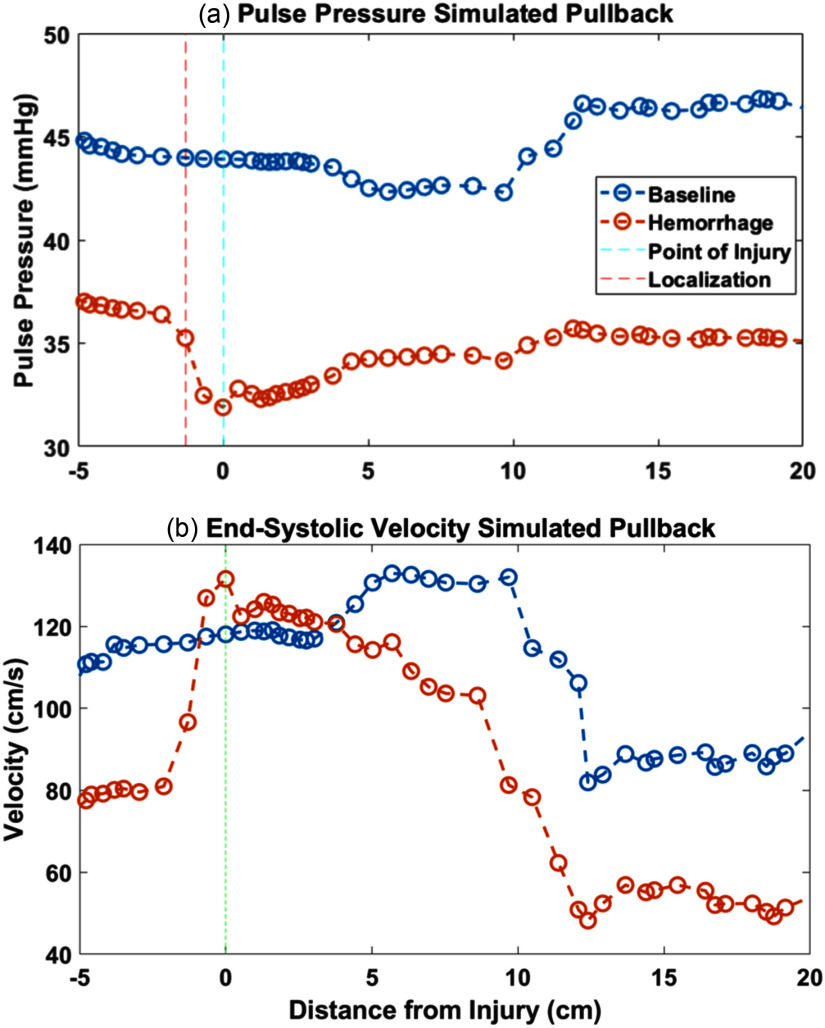
(a) PP with localization and (b) ESV during simulated pullback, comparing 800 ml/min hemorrhage case and baseline.

As shown in Fig. 8(a), the simulation exhibits a PP decrease of 5 mmHg at the injury point, consistent with the in vivo measurements. The simulation also shows a velocity increase of 55 cm/s in Fig. 8(b), which cannot be compared to the in vivo experiments since velocity was not measured. These changes are not evident in the baseline simulations, confirming that the changes are specific to the injury. The simulation also shows a PP increase and ESV decrease in the vicinity of 10 cm, for both the baseline and injury cases. These changes coincide with the bifurcations of the major abdominal aortic branch arteries. Applying the localization algorithm used in the in vivo experiment identifies the point of maximum negative change at 1.3 cm before the known location of the injury in the model.

## IV. Discussion

Results from a combination of in vivo measurements, in silico modeling, and algorithm development indicate the potential to localize an aortic hemorrhage with a pressure-sensing catheter.

The algorithm located the hemorrhage with a mean error of 0.05 cm and a standard deviation of 3.46 cm. With further refinement, the algorithm may prove sufficiently accurate to stop the hemorrhage by placing a covered stent. The algorithm is well suited to real-time implementation. The algorithm requires pulling the catheter back past the hemorrhage site in order to locate the hemorrhage, i.e., the algorithm is noncausal. Once the site is located, the catheter would be pushed back to the hemorrhage site to perform an intervention and assess results.

The algorithm is also able to differentiate between a non-injury (baseline) and injury case from the PP pullback profile. Baseline pullback data consistently show an increasing PP profile, e.g., Fig. 5(a), whereas injury pullback data show a decreasing PP profile. The method can thus confirm noninvasive detection of internal bleeding [19], [20], [23].

The in silico model displayed a similar drop in pulse pressure across the hemorrhage site as was measured in vivo. The model also displayed an increase in end-systolic velocity, suggesting that flow sensing would also aid in localizing the injury site.

Other work located a hemorrhage in a porcine femoral artery from a reduced difference in systolic and diastolic velocity, resulting in a decreased resistivity index [16], [17]. The resistivity index was measured noninvasively using Doppler ultrasound. Since aortic flow waveforms will be more straightforward to measure endovascularly, we plan to assess effectiveness of the resistivity index in future work. The resistivity index has been principally used outside of the setting of hemorrhage, for detecting renal stenoses and assessing organ damage [24]. The instantaneous wave-free ratio (iFR) [25], [26] is another technique for quantifying coronary artery stenosis, based on a drop in pressure proximal-to-distal from the stenosis, measured during the wave-free period of diastole [27]. Since stenosis and vasospasm both constrict arterial flow, we analyzed this method with our in vivo data but did not find it to be effective for locating the hemorrhage and vasospasm.

This work has several limitations. The in vivo measurements were performed on a relatively small number of five pigs, with high hemorrhage rates that are not clinically relevant. While these data serve to indicate the concept, future experiments will expand the number of test subjects and will broaden the range of hemorrhage rates. Future experiments will also quantify additional aspects of the compensatory response, such as the physical length of the vasospasm and duration. The estimated algorithm accuracies are subject to the 1 s quantization in truth time stamps and to variations in the manual pullback rate, estimated as about 1 cm/s. Future work will utilize motor-controlled pullback as has been performed for iFR [27] and will measure flow waveforms in addition to pressure. Another limitation of the porcine experiments is that the animals were anesthetized with isoflurane, which is common for porcine hemorrhage models but which can cause vasodilation and hypotension. However, these effects are dose-dependent and the investigators kept the dose at the lowest level possible while keeping the animals fully anesthetized.

The in silico modeling was based on a human subject in order to assess whether pulse pressure would be similarly informative as for porcine measurements. Additional in silico modeling will also include porcine cases.

The ability to accurately analyze and detect arterial hemorrhage is critical in various clinical settings, particularly in cases of acute traumatic arterial bleeding. This includes both blunt and penetrating injuries, where rapid identification and control of hemorrhage can be lifesaving. Additionally, bleeding assessment is essential for post-procedural monitoring following invasive endovascular interventions, such as iliac, aortic, or subclavian artery stenting. In these cases, prompt confirmation of vessel integrity is necessary to rule out complications, including bleeding at the edges of an arterial stent or hemorrhage in high-flow vessels, where rapid contrast washout may limit the effectiveness of conventional angiographic analysis.

Beyond traditional hospital settings, future applications of this bleeding analysis technology could be particularly impactful in combat and austere environments, where immediate access to a trauma system or vascular surgeon is not feasible. In such scenarios, early and precise identification of hemorrhage is essential for guiding temporizing interventions, such as the placement of a Resuscitative Endovascular Balloon Occlusion of the Aorta (REBOA) device by trained non-surgeon healthcare providers. The ability to rapidly localize vascular injury in these settings may significantly improve survival by enabling early hemorrhage control and optimizing patient stabilization until definitive surgical care is available. To support this capability, technology is also being advanced to allow non-specialists to more readily insert a catheter in the femoral artery [28].

Additionally, the ability to exclude major arterial hemorrhage is equally critical for triage and evacuation decisions, particularly in resource-limited environments where advanced imaging modalities, such as CT, may be unavailable, limited, or in high demand. Accurate assessment of bleeding risk can inform transport modality choices (e.g., land vs. air evacuation) and prioritize patients based on the urgency of intervention, ultimately improving outcomes in both military and civilian trauma patient scenarios.

A principal objective of our future work will be to broaden endovascular hemorrhage localization beyond aortic hemorrhage, to the vascular territories of primary aortic branch arteries. While localizing aortic hemorrhage is important in its own right and could save lives [29], if primary aortic branch arteries with hemorrhage can be detected, there is the potential for more-localized embolization, as opposed to the current aortic occlusion (REBOA).

## V. Conclusion

This paper presents: 1) a novel experimental protocol that explores high-fidelity endovascular localization using arterial blood pressure waveform feature data and 2) a high-resolution simulated model that validates the phenomenon observed in the porcine experiments. The results show the ability to accurately localize an injury within less than 5 cm, a significant improvement over the torso quadrant methods currently employed by methods such as the FAST exam and devices such as REBOA. Future work will explore adding endovascular blood velocity and flow measurements on a porcine model with injuries induced in an organ to see if there are detectable signals in the aorta or near the entrance to a branch artery of the afflicted organ system.

## Supplementary Materials

The supplementary materials provide more details on the signal processing pipeline for feature extraction, the simulated model, and porcine subject summary statistics.

Supplementary Materials

## Author Contributions

BAT and MWC conceptualized the paper. MWC, TGH, and JMR designed and executed the animal experiments. RLT and MCJ developed the computational model and produced the in silico data. SHA and BAT performed the formal analysis, developed the localization algorithm, and drafted the first draft of the manuscript. All authors subsequently contributed to reviews and edits.

All authors have read and approved the final version of the manuscript.

## Ethics Statement

All authors confirm that this research adheres to the ethical guidelines and standards set by IEEE Open Journal of Engineering in Medicine and Biology (OJEMB). Where applicable, ethical approval was obtained from the relevant institutional review board (IRB) or ethics committee, and all procedures involving human participants or animals were conducted in accordance with the ethical standards of the Declaration of Helsinki or other relevant ethical guidelines. The research does not contain any material that violates ethical norms, and every effort has been made to ensure the integrity, transparency, and reproducibility of the study.

## Conflicts of Interest

The authors declare that there are no conflicts of interest related to this study.
