# Supplementary material for: Endovascular Localization of Aortic Injury in a Porcine Model
Source: IEEE Open J Eng Med Biol. 2025 Apr 2;6:425–31. doi: 10.1109/OJEMB.2025.3556987 (PMC12250867; doi:10.1109/OJEMB.2025.3556987)
Supplement: Supplementary Materials [file supp1-3556987.docx]

**Supplementary Materials**

Endovascular Localization of Aortic Injury in a Porcine Model

Saaid H. Arshad, Ryan L. Touzjian, Matthew C. Jones, Brian A. Telfer, Jason M. Rall, Theodore G. Hart, Marlin W. Causey

# Materials and Methods


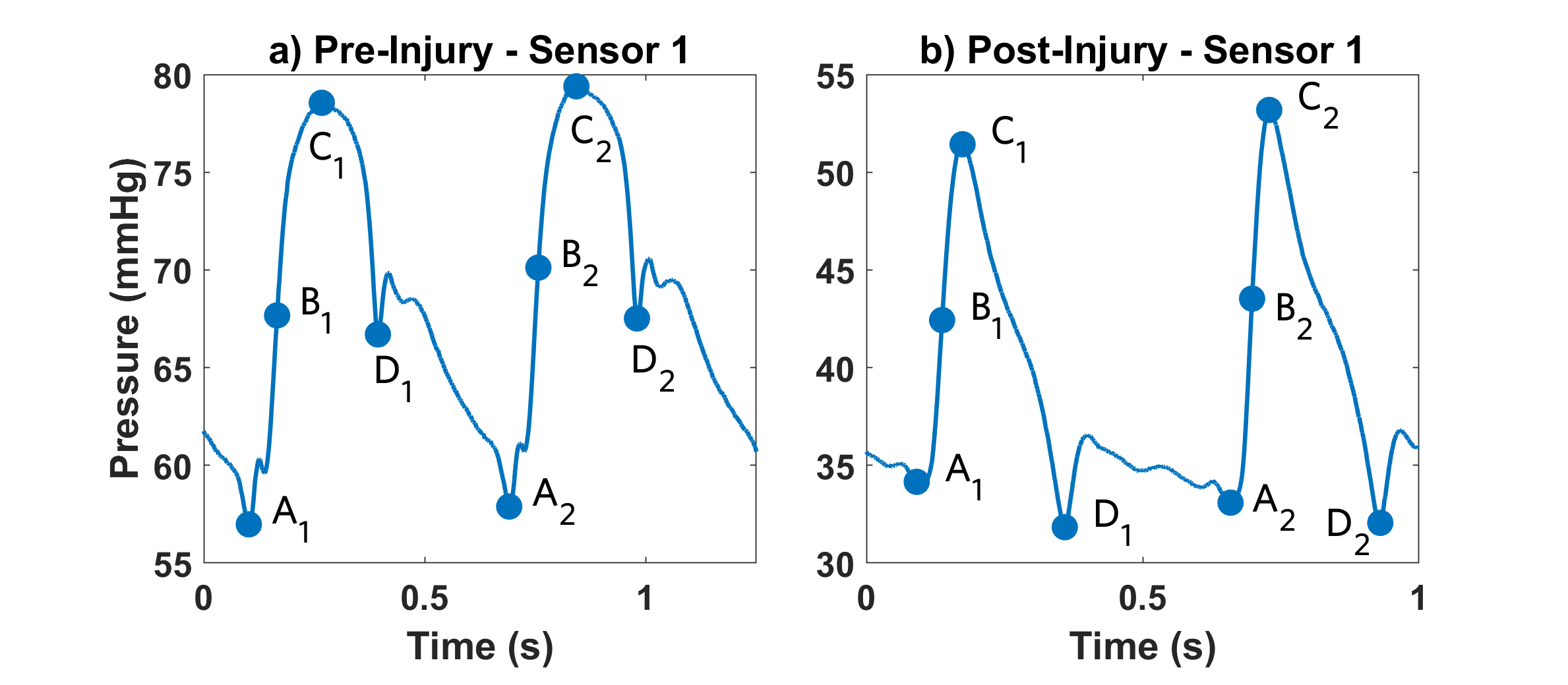


| Feature | Description |  |
| --- | --- | --- |
|  | Name | Formula ^a^ |
| PPI | Peak to Peak Interval | $t_{A_{1}}-t_{A_{2}}$ |
| HRV | Heart Rate Variability | $RMSSD$ of PPI for 10 beats |
| HRDN | Half-Rise to Dicrotic Notch | $t_{B}-t_{D}$ |
| SBP | Systolic Blood Pressure | $W_{C}$ |
| DBP | Diastolic Blood Pressure | $W_{A}$ |
| PP | Pulse Pressure | $W_{C}-W_{A}$ |
| PA | Pulse Area | $\int_{A_{1}}^{A_{2}} W dt$ |
| IPA | Inflection Point Area | $\int_{D_{1}}^{A_{2}} W dt \div\int_{A_{1}}^{D_{1}} W dt$ |
| SI | Shock Index | $\frac{60}{PPI\cdot SBP}$ |

$a. W_{x}$ is the ABP signal value at point $x$ and $t_{x}$ is the time at point $x$. RMSSD: Root mean squared of successive differences.

Fig. 1. Two sample endovascular pressure pulses are shown from subject 1654 a) before and b) after the injury is induced. Automatically computed landmark points are plotted for (A) start of pulse, which is also DBP, (B) systolic half-rise, (C) systolic peak (SBP), and (D) dicrotic notch. The corresponding table below describes the various features computed.

## In vivo Porcine Hemorrhage Model

Initial analgesia and sedation were accomplished using intramuscular injections of 4.4 mg/kg tiletamine/zolazepam, 2.2 mg/kg ketamine, and 0.01 buprenorphine. The animals were then intubated, mechanically ventilated, and had anesthesia maintained using inhaled isoflurane at a concentration of 1.0% to 3.5% throughout the protocol. A midline neck dissection was performed on each animal with cannulation of the bilateral carotid arteries. The femoral arteries were accessed percutaneously.

## Arterial Blood Pressure Waveform Signal Processing

### Preprocessing

For calculating pulse features such as the pulse peak and trough, the preprocessing and feature-extraction protocols used in previous work [1, 2] were utilized. Summarizing those steps, the endovascular pressure recordings were first smoothed by a 512th order zero-phase finite impulse response lowpass filter with 6 Hz cutoff frequency (MATLAB, Natick, MA, USA). First and second derivatives were approximated as finite differences of the denoised ABP traces. Signals and derivatives were z-scored within a 2-s trailing window to detrend, remove baseline drift, and standardize amplitude scaling.

### Feature Extraction

Fiducial points corresponding to the landmarks in Fig. 1 were identified based on peak finding (MATLAB 2023b) and empirical relationships between the ABP signal and its derivatives, as described in [3] and applied in [1,2]. Features describing the morphology of each ABP pulse were then computed using the formulas in the Table in Fig. 1 [3]. These features reflect interpretable physiological correlates such as cardiac output, autonomic function, and peripheral vascular resistance [1,2]. The feature set also includes standard vital sign features such as heart rate (HR), systolic blood pressure (SBP), and diastolic blood pressure (DBP).

Since the ABP waveform is modulated by breathing, the derived features were also affected by breathing, which introduced noise in the final localization step. To remove the effects of breathing on the extracted feature values, the feature vectors were further filtered by an 11th order low-pass FIR filter with a 0.15 Hz cutoff to ensure removal of any respiratory interference.

### Outlier Rejection

Outlier values for each feature were detected using a moving median filter within a centered, 20-heartbeat window. Data points greater than three scaled median absolute deviations (MAD) away from the window median were removed. Scaled MAD was calculated as 1.5 *median(|A-median(A)|) for each sequence, A, of local feature values.

DISTRIBUTION STATEMENT A. Approved for public release. Distribution is unlimited. This material is based upon work supported by the Defense Health Agency, Combat Casualty Care Research Program (CCCRP) under Air Force Contract No. FA8702-15-D-0001. Any opinions, findings, conclusions or recommendations expressed in this material are those of the author(s) and do not necessarily reflect the views of the Defense Health Agency.

## Computational Aorta Model

The computational model uses the SimVascular software package [3].

The workflow of this software starts with a CT scan of the subject, from which a geometric model of the arterial system is produced. The blood flow into the aorta from the heart is modeled using a waveform of volumetric flow over time. The geometric model captures the arterial system from the heart outflow, through the aortic root to the femoral arteries, and includes partial lengths of the major branch arteries. The domain terminates with outlet surfaces at the chosen “end” of each branch artery. The behavior of the flow out of the computational domain through these outlet surfaces is governed by 3-element Windkessel [4] models, which are simplified 0-D representations of downstream vasculature. In the modeled computational domain, a 3D incompressible laminar Navier-Stokes Computational Fluid Dynamics (CFD) solver governs the movement of blood, and it is coupled to a thin-walled structural model representing the compliance of the aortic wall. This coupled-momentum method [5] solves the flow behavior interdependently with the deformation of the physical wall, ensuring both sets of physics are taken into account. The result of this simulation is a 3D flow-field with pressure and velocity predictions for every point in space and time within the geometrically modeled aorta and branch arteries. Spatial and temporal wall deformation is also computed.

The baseline model was modified to represent the hemorrhage and compensatory response, including the vasospasm. The hemorrhage was modeled by adding a short (1 - 2 vessel diameters long) tube in the geometric domain, with a pressure resistance outflow condition. The resistance was tuned to adjust level of blood loss through this outlet, which is governed by the resistance equation, where blood flow is proportional to pressure at the outlet. It is assumed the hemorrhaging flow is entering a zero-pressure region in the body. The vasospasm was modeled as a local vasoconstriction with a 23% diameter reduction, consistent with in vivo measurements (Main Section IIA.2). To model the compensatory response to hemorrhage, resistance in the Windkessel models was increased by 33%, heart rate was increased by 20% and stroke volume was decreased by 40%, based on in vivo measurements for approximately 20% blood loss in swine [6] and baboons [7].

Table II: Flow distribution among branch arteries (values scaled to account for flow into branches not modeled in MIT Lincoln Laboratory simulation)

|  | **Flow Per Cardiac Cycle [%]** | | |
| --- | --- | --- | --- |
| **Branch Artery** | **Benim [16]** | **Xiao [17]** | **MIT LL** |
| Brachiocephalic | 15.8 | 10.6 | 17.6 |
| Left Common Carotid | 7.9 | 2.2 | 6.6 |
| Left Subclavian | 7.9 | 8.5 | 11.0 |
| Celiac | 15.8 | 13.5 | 14.9 |
| Superior Mesenteric | 10.5 | 16.3 | 10.3 |
| Right Renal | 10.5 | 13.4 | 10.5 |
| Left Renal | 10.5 | 13.4 | 10.5 |
| Right Common Iliac | 10.5 | 11.0 | 9.3 |
| Left Common Iliac | 10.5 | 11.0 | 9.3 |

The computational model was initialized with a vascular geometric model, an aortic inflow waveform, Windkessel parameters, and vessel wall mechanical properties. A rigid-wall vascular model of a healthy 21-year-old female was selected from the Vascular Model Repository [8]. This model [9] includes an inflow waveform and a set of outflow Windkessel parameters. The aortic inflow waveform (see Fig. 2) is based on reference [10] and was scaled in both time and magnitude to achieve desired heart rate and cardiac output.

The 3-element Windkessel model parameters, representing distal vasculature, were previously tuned to match flow distribution among the various outlets as described in the literature [9].

The vessel wall mechanical properties were added to simulate wall compliance and are estimated based on data from Cuomo et. al. [11] and Snyder [12]. Wall thickness and stiffness were kept uniform throughout the model as specified in Table I.

To run the Simvascular software, it was installed on the MIT Lincoln Laboratory Supercomputing Center [13]. The computational mesh was designed with 11 million elements.

Computational solver settings included a time step of 0.001 seconds, and each simulation was solved to a 10 second duration, notionally capturing 10 cardiac cycles when HR = 60 BPM. Only results from the last cardiac cycle are used, to allow initial transients to disappear and flow to reach a periodic state.

To confirm that the computational model generates plausible results, three checks were conducted. First, the change in ascending aorta vessel diameter during the cardiac cycle was assessed. The model resulted in a change of 1.4mm, which is consistent with an expansion of 1.78±0.44mm described for an age range of 29-76 years old [14]. Second, the aortic pressure wave velocity was assessed, with the model’s 575 cm/s velocity consistent with a range of 550 and 780 cm/s measured for healthy humans at similar ages [15]. Third, the modeled flow distributions through the primary branch arteries were found to be similar to those reported in other literature sources (see Table II) [16,17].

Table I: Characterization of the mechanical aspects of the arterial walls.

| **Location** | **Thickness [cm]** | **Young’s Modulus [Pa]** | **Poisson Ratio** | **Shear Constant** | **Density [g/cm3]** |
| --- | --- | --- | --- | --- | --- |
| All Vessel Walls | 0.17 | 500,000 | 0.5 | 0.83 | 1.0 |

Table IV: Numbers of static and pullback measurements across the five pigs.

| Subject | Baseline Pullbacks | Injury Pullbacks | Baseline Static | Injury Static |
| --- | --- | --- | --- | --- |
| 1625  4/27/2023 | 2 | 3 | 0 | 1 |
| 1626  4/27/2023 | 2 | 6 | 0 | 3 |
| 1654  5/24/2023 | 4 | 2 | 2 | 2 |
| 1655  5/24/2023 | 1 | 2 | 2 | 2 |
| 1672  5/31/2023 | 3 | 2 | 2 | 1 |
| Total | 12 | 15 | 6 | 9 |


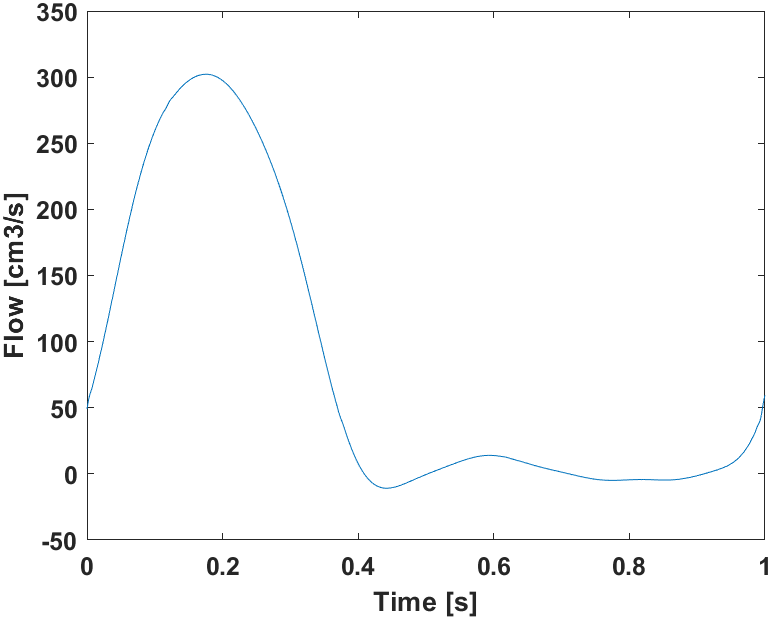


Fig. 2: An example of the model’s blood flow profile pumped by the heart for a single beat.

Table III: Weight and baseline characteristics of each pig. EOS: end of surgery; EtCO2: End-tidal carbon dioxide; MAP: Mean arterial pressure; SBP: systolic blood pressure

| Subject | Weight (kg) | Injury to EOS  (minutes) | Baseline SBP  (mmHg) | Baseline MAP (mmHg) | Baseline HR (bpm) | Baseline EtCO2 (mmHg) | Blood Loss (mL) |
| --- | --- | --- | --- | --- | --- | --- | --- |
| 1625  4/27/2023 | 88 | 7.92 | 84.1 | 66.1 | 103 | 41 | 1850 |
| 1626  4/27/2023 | 82 | 16.92 | 80.1 | 61.4 | 85 | 42 | 1600 |
| 1654  5/24/2023 | 79 | 5.87 | 73.6 | 56.7 | 85 | 42 | 2400 |
| 1655  5/24/2023 | 75 | 4.3 | 81.6 | 56.9 | 155 | 45 | 1700 |
| 1672  5/31/2023 | 89 | 5.77 | 89.2 | 67.9 | 84 | 42 | 2500 |

# Results

Fig. 3: Pressure traces over the entire experiment for each pig.

Fig. 3 (continued): Pressure traces over the entire experiment for each pig. Zero minutes indicates when the injury was induced. The large step changes in the pressure observed in each experiment occur when the CODA balloon is inflated to block blood flow to allow the creation of the aortic injury.

Table V: The results of the initial ROC analysis on the ability of each feature to classify ABP pulse data as proximal or distal to the point of injury based on the injury point time stamp observed experimentally. See Figure 1 in this document for feature acronyms. The feature with highest area under the ROC curve (AUC) is highlighted in green.

| Feature | AUC | Normalized Feature AUC |
| --- | --- | --- |
| PPI | 0.5 | 0.54 |
| HRV | 0.67 | 0.74 |
| HRDN | 0.56 | 0.6 |
| SBP | 0.74 | 0.87 |
| DBP | 0.55 | 0.58 |
| PP | 0.93 | 0.95 |
| PA | 0.61 | 0.66 |
| IPA | 0.61 | 0.53 |
| SI | 0.71 | 0.72 |

Table III reports the weight, pressures, HR, end-tidal carbon dioxide, blood-loss, and time from injury to end-of-surgery for each pig used for the experiments. Table IV details the number of static and pullback measurements collected per pig during baseline and injury. A total of 42 measurements were made for the five pigs, with each measurement approximately 30s in duration.

Table V shows the results of the initial ROC analysis to determine the ability of each feature to classify ABP data as either proximal or distal of the injury point. The pressure-based features, namely PP and SBP had the highest areas-under-the-curve (AUCs) before and after normalization. Since PP as a raw feature performs the best, the main analysis focuses on PP.

Figure 3 shows the pressure traces of all sensors over the entire duration of the experiment with each pig beginning from baseline recordings and inflation of the catheter balloon. Figure 4 shows a snapshot of the LabChart software used to display the real-time acquired pressures signals from the Millar catheter.


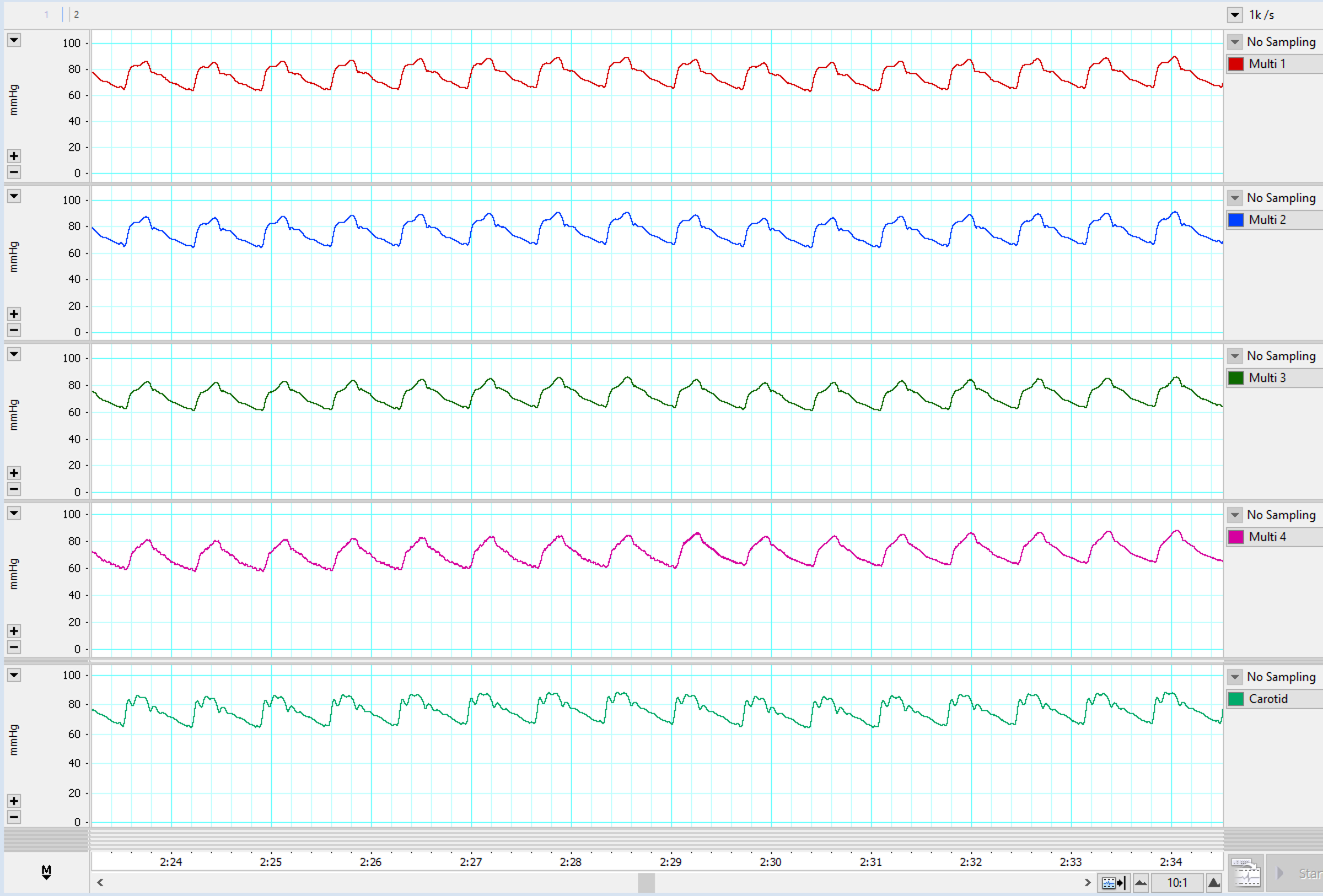


Fig. 4: Snapshot of LabChart signals from subject 1672. Data from sensors one through four along with a reference measurement in the carotid artery are displayed.

References

1. Gupta, J. F., Telfer, B. A. & Convertino, V. A. Feature Importance Analysis for Compensatory Reserve to Predict Hemorrhagic Shock. *2022 44th Annu Int Conf IEEE Eng Medicine Biology Soc EMBC* 00, 1747–1752 (2022). Available: https://pubmed.ncbi.nlm.nih.gov/36086009/
2. Gupta, J. F., Arshad, S. H., Telfer, B. A., Snider, E. J. & Convertino, V. A. Noninvasive Monitoring of Simulated Hemorrhage and Whole Blood Resuscitation. *Biosensors* 12, 1168 (2022). Available: https://pubmed.ncbi.nlm.nih.gov/36551134/
3. Elgendi, M. On the Analysis of Fingertip Photoplethysmogram Signals. *Curr Cardiol Rev* 8, 14–25 (2012). Available: <https://pubmed.ncbi.nlm.nih.gov/22845812/>
4. Updegrove, A. *et al.* SimVascular: An Open Source Pipeline for Cardiovascular Simulation. *Ann. Biomed. Eng.* 45, 525–541 (2017). Available: https://pubmed.ncbi.nlm.nih.gov/27933407/).
5. Figueroa, C. A., Vignon-Clementel, I. E., Jansen, K. E., Hughes, T. J. R. & Taylor, C. A. A coupled momentum method for modeling blood flow in three-dimensional deformable arteries. *Comput. Methods Appl. Mech. Eng.* 195, 5685–5706 (2006). Available: https://www.sciencedirect.com/science/article/pii/S004578250500513X
6. Toung, Reilly, P. M., Fuh, K. C., Ferris, R. & Bulkley, G. B. Mesenteric Vasoconstriction in Response to Hemorrhagic Shock. *SHOCK* 267–273 (2000). Available: https://pubmed.ncbi.nlm.nih.gov/10774614/
7. Hinojosa-Laborde, C. *et al.* Validation of lower body negative pressure as an experimental model of hemorrhage. *J Appl Physiol* 116, 406–415 (2014). Available: https://pubmed.ncbi.nlm.nih.gov/24356525/
8. (Segers, P. *et al.* Towards a consensus on the understanding and analysis of the pulse waveform: Results from the 2016 Workshop on Arterial Hemodynamics: Past, present and future. *Artery Res.* 18, 75–80 (2017). Available: <https://pmc.ncbi.nlm.nih.gov/articles/PMC5470638/>)
9. Wilson, N. M., Ortiz, A. K. & Johnson, A. B. The Vascular Model Repository: A Public Resource of Medical Imaging Data and Blood Flow Simulation Results. *J. Méd. Devices* 7, 040923 (2013). Available: https://pmc.ncbi.nlm.nih.gov/articles/PMC4023857/
10. Updegrove, A. *et al.* SimVascular: An Open Source Pipeline for Cardiovascular Simulation. *Ann. Biomed. Eng.* 45, 525–541 (2017). Available: https://pubmed.ncbi.nlm.nih.gov/27933407/
11. Cuomo, F. *et al.* Effects of age-associated regional changes in aortic stiffness on human hemodynamics revealed by computational modeling. *PLoS ONE* 12, e0173177 (2017). Available: https://pmc.ncbi.nlm.nih.gov/articles/PMC5333881/
12. Snyder, M. F., Rideout, V. C. & Hillestad, R. J. Computer modeling of the human systemic arterial tree. *J. Biomech.* 1, 341–353 (1968). Available: https://pubmed.ncbi.nlm.nih.gov/16329438/
13. Reuther, A. *et al.* Interactive Supercomputing on 40,000 Cores for Machine Learning and Data Analysis. *2018 IEEE High Perform. extreme Comput. Conf. (HPEC)* 00, 1–6 (2018). Available: https://arxiv.org/abs/1807.07814
14. Huang, J. *et al.* Comparison of dynamic changes in aortic diameter during the cardiac cycle measured by computed tomography angiography and transthoracic echocardiography. *J. Vasc. Surg.* 69, 1538–1544 (2019). Available: <https://pubmed.ncbi.nlm.nih.gov/31010518/>
15. O’Rourke, M. F., Staessen, J. A., Vlachopoulos, C., Duprez, D. & Plante, G. érard E. Clinical applications of arterial stiffness; definitions and reference values. *Am. J. Hypertens.* 15, 426–444 (2002). Available: https://pubmed.ncbi.nlm.nih.gov/12022246/
16. Benim, A. C. *et al.* Simulation of blood flow in human aorta with emphasis on outlet boundary conditions. *Appl. Math. Model.* 35, 3175–3188 (2011). Available: https://www.sciencedirect.com/science/article/pii/S0307904X10005214
17. Xiao, N., Alastruey, J. & Figueroa, C. A. A systematic comparison between 1‐D and 3‐D hemodynamics in compliant arterial models. *Int. J. Numer. Methods Biomed. Eng.* 30, 204–231 (2014). Available: https://pubmed.ncbi.nlm.nih.gov/24115509/
